# Transcriptomic Analysis Reveals the Molecular Mechanisms of Drought-Stress-Induced Decreases in *Camellia sinensis* Leaf Quality

**DOI:** 10.3389/fpls.2016.00385

**Published:** 2016-03-30

**Authors:** Weidong Wang, Huahong Xin, Mingle Wang, Qingping Ma, Le Wang, Najeeb A. Kaleri, Yuhua Wang, Xinghui Li

**Affiliations:** College of Horticulture, Nanjing Agricultural UniversityNanjing, China

**Keywords:** *Camellia sinensis*, drought stress, quality, secondary metabolite, RNA-Seq, molecular mechanisms

## Abstract

The tea plant [*Camellia sinensis* (L.) O. Kuntze] is an important commercial crop rich in bioactive ingredients, especially catechins, caffeine, theanine and other free amino acids, which the quality of tea leaves depends on. Drought is the most important environmental stress affecting the yield and quality of this plant. In this study, the effects of drought stress on the phenotype, physiological characteristics and major bioactive ingredients accumulation of *C. sinensis* leaves were examined, and the results indicated that drought stress resulted in dehydration and wilt of the leaves, and significant decrease in the total polyphenols and free amino acids and increase in the total flavonoids. In addition, HPLC analysis showed that the catechins, caffeine, theanine and some free amino acids in *C. sinensis* leaves were significantly reduced in response to drought stress, implying that drought stress severely decreased the quality of *C. sinensis* leaves. Furthermore, differentially expressed genes (DEGs) related to amino acid metabolism and secondary metabolism were identified and quantified in *C. sinensis* leaves under drought stress using high-throughput Illumina RNA-Seq technology, especially the key regulatory genes of the catechins, caffeine, and theanine biosynthesis pathways. The expression levels of key regulatory genes were consistent with the results from the HPLC analysis, which indicate a potential molecular mechanism for the above results. Taken together, these data provide further insights into the mechanisms underlying the change in the quality of *C. sinensis* leaves under environmental stress, which involve changes in the accumulation of major bioactive ingredients, especially catechins, caffeine, theanine and other free amino acids.

## Introduction

Tea plant [*Camellia sinensis* (L.) O. Kuntze] is an important perennial, evergreen, woody crop that is grown worldwide, and its young leaves are processed to prepare a popular non-alcoholic beverage known as “tea.” *C. sinensis* plants experienced the effects of various abiotic stresses during their lifecycle, such as drought stress (Das et al., 2012), temperature stress, salinity stress (Li et al., 2010), heavy-metal stress (Basak et al., 2001), and soil nutrient deficiency (Upadhyaya and Panda, 2013). Among these stresses, drought stress is an important factor that significantly constrains the yield and quality of tea products, and damage caused by drought has become increasingly frequent and unpredictable due to global climate changes, especially growing water scarcity (Zhou et al., 2014). At present, research on the effects of drought stress in *C. sinensis* plants is focused on the mechanisms underlying the stress response, which includes morphological, physiological, and molecular changes (Upadhyaya et al., 2013). For example, Zhou et al. (2014) and Upadhyaya et al. (2013) reported that the amounts of H_2_O_2_, chlorophyll, proline, and MDA and the activities of enzymes such as SOD, CAT, POX, GR, and PPO changed in *C. sinensis* plants under drought stress. In addition, differentially expressed genes related to the drought response were identified using suppression subtractive hybridization and cDNA-AFLP technology (Das et al., 2012; Gupta et al., 2013). Furthermore, two-dimensional electrophoresis identified proteins that are differentially expressed in response to drought stress (Lin et al., 2014). In contrast, there are only few studies have focused on the effects of drought stress on *C. sinensis* leaf quality, especially the changes in its main bioactive ingredients, such as catechins, caffeine, theanine and other free amino acids.

The production of secondary metabolites in *C. sinensis* plants contribute to the rich flavors, clean taste, and nutrient content of tea, and these metabolites are known to be beneficial to human health (Li et al., 2015a). Among them, polyphenols (especially catechins), caffeine, and theanine are the most important constituents that determine the quality of *C. sinensis* leaves, which further determines the quality of tea products (Liang et al., 1990). Previous studies have shown that a poor growth environment strongly reduces the quality of *C. sinensis* leaves by changing the amounts of polyphenols, caffeine, and theanine (Ahmed et al., 2014; Zhang et al., 2014). For example, Zheng et al. (2008) reported that excessive UV-B irradiation suppressed the accumulation of tea catechins, and Zhang et al. (2014) demonstrated that the accumulation of individual catechins, caffeine and free amino acids was influenced by light intensity and temperature. Similarly, the amounts of bioactive ingredients in *C. sinensis* leaves, such as polyphenols, caffeine and free amino acids, were found to decrease in response to an extreme acid-rain environment (Duan et al., 2012). In addition, preliminary experimental results showed that drought stress affected the accumulation of polyphenols and individual bioactive ingredients, which decreased the quality of *C. sinensis* leaves (Jeyaramraja et al., 2003; Chen et al., 2010). However, direct and detailed evidence of these negative impacts is limited. On the other hand, the molecular mechanisms of major metabolic pathways in *C. sinensis* have been a focus of study, and efforts have been made to identify the key genes involved in several major metabolic pathways in *C. sinensis* (Li et al., 2015a). For example, Shi et al. (2011) elucidated the gene network responsible for the regulation of the secondary metabolite biosynthetic pathways in *C. sinensis* using high-throughput Illumina RNA-Seq technology, especially the flavonoid biosynthesis pathway (namely, the catechins biosynthesis pathway in *C. sinensis*), the caffeine biosynthesis pathway and the theanine biosynthesis pathway. Recently, Li et al. (2015a) analyzed the gene expression profiles related to secondary metabolic pathways in different tissues at different developmental stages in *C. sinensis*, which further revealed how secondary metabolic pathways are regulated during plant development and growth cycles. However, the molecular mechanisms underlying the effects of environmental stress (especially drought stress) on the accumulation of secondary metabolites in *C. sinensis*, such as catechins, caffeine, theanine and other free amino acids, remain unknown.

In the present study, we investigated the effects of drought stress on the phenotype, physiological characteristics and major bioactive ingredients accumulation in *C. sinensis* leaves, including the total polyphenol, flavonoid and free amino acid content. In addition, the changes in the levels of catechins, caffeine, theanine and other amino acids in *C. sinensis* leaves were detected by HPLC after treatment with drought stress. Furthermore, to investigate the molecular mechanisms of decrease in quality of tea leaves under drought stress, differentially expressed genes (DEGs) related to amino acid metabolism and secondary metabolism in *C. sinensis* plants in response to drought stress, especially genes associated with the catechin, caffeine and theanine biosynthesis pathways were identified analyzed. These data were used to explore the molecular mechanisms underlying the changes in the accumulation of the main bioactive ingredients that occur in response to drought stress and influence leaf quality in *C. sinensis*.

## Materials and methods

### Plant materials

Two-year-old tea plants [*Camellia sinensis* (L.) O. Kuntze cv. “*Longjingchangye*”] were pre-incubated under normal conditions (25 ± 1°C, 12-h light/12-h dark cycle) for 2 weeks in an artificial climate chamber. The drought stress assays were then carried out using 20% (w/v) polyethylene glycol (PEG) 6000 with all of the other environmental conditions remaining constant. The first and second tender leaves from about 120°C. *sinensis* plants were randomly collected at several time points (0, 2, 12, 24, and 48 h) under control or stress conditions, and the samples were immediately frozen in liquid nitrogen and stored at −80°C for further analysis. Additionally, following 0, 2, and 5 days of treatment, *C. sinensis* leaves were randomly collected, freeze-dried and ground to a fine powder to analyze the effects of drought stress on the metabolism of the main chemical components.

### Determination of chlorophyll, malondialdehyde, and relative water content

During the period of drought stress, treated leaves were randomly sampled at 0, 2, and 5 days from about 120°C. *sinensis* plants after the application of 20% PEG 6000, and the samples were used to determine the physiological characteristics. Relative water content (RWC) was measured to detect the effects of drought stress on *C. sinensis* plants according to Upadhyaya et al. (2008). The content of chlorophyll and malondialdehyde (MDA) in the leaves were determined by spectrophotometer method developed by Knudson et al. (1977) and Dhindsa et al. (1981), respectively.

### Determination of total polyphenol, flavonoid, and free amino acid content

The total polyphenols were extracted from the powdered leaf samples in 70% (v/v) methanol at 70°C, and the content was assessed using a UV-5200 spectrophotometer (METASH, China) at 765 nm according to the Folin-Ciocalteu method described by Li et al. (2015b). The total flavonoids and free amino acids were extracted in deionized water at 100°C, and the flavonoid content was determined according to the AlCl_3_ method (Lin and Tang, 2007) and ninhydrin coloration method (Zheng et al., 2015).

### Quantification of catechins and caffeine by HPLC

The catechin and caffeine content was determined by high-performance liquid chromatography (HPLC) according to Chen et al. (2015) with some modifications. Briefly, 0.2 g of powdered leaves was extracted with 10 mL of 70% (v/v) methanol at 70°C for 20 min, and the extract was then filtered through a 0.45-μm Millipore filter before being injected into an Shimadzu LC-20A HPLC system (Shimadzu, Japan). A 5-μL volume of filtrate was injected into the HPLC system and analyzed on an Inertsil ODS-SP C18 analytical column (250 × 4.6 mm i.d., 5 μm nominal particle size). A solution containing 9% (v/v) methyl cyanide, 2% (v/v) acetic acid, and 0.02% (m/v) EDTA was used as mobile phase A, and mobile phase B consisted of 80% (v/v) methyl cyanide, 2% (v/v) acetic acid and 0.02% (m/v) EDTA. The samples were eluted at 35°C at a flow-rate of 1 mL/min, and a continuous eluent gradient was adopted to enhance peak separation. The absorbance at 278 nm was used to monitor peak intensities in real-time, and the peaks were identified by comparing the retention times for the sample to those of authentic standards. Authentic standards for epigallocatechin gallate (EGCG, ≥ 95%), epicatechin gallate (ECG, ≥ 98%), gallocatechin (GC, ≥ 98%), epigallocatechin (EGC, ≥ 95%), catechin (Cat, ≥ 97%), epicatechin (EC, ≥ 98%), gallocatechin gallate (GCG, ≥ 98%), and caffeine (≥ 95%) were purchased from Sigmae-Aldrich (St. Louis, MO, USA).

### Extraction and quantitative analysis of theanine and other amino acids

Theanine was extracted from the samples with deionized water for 45 min in a water bath at 80°C, and the extract was filtered through a 0.45-μm Millipore filter before HPLC analysis. The theanine was then detected using an Shimadzu LC-20A HPLC system (Shimadzu, Japan) according to the method described by Tai et al. (2015). In addition, other amino acids were extracted as described by Wan et al. (2015) with modifications, and 17 common amino acids were identified using an L-8900 automatic amino acid analyzer (Hitachi, Japan).

### RNA extraction, library construction, and RNA-Seq

Total RNA from *C. sinensis* leaves was extracted using RNAiso Plus (TaKaRa, Japan). The integrity and quality of RNA was measured using a 2100 Bioanalyzer RNA Nano chip device (Agilent, Santa Clara, CA, USA) and a NanoDrop ND-1000 spectrophotometer (NanoDrop, Wilmington, DE). Equal amounts of RNA from three biological replicates were pooled prior to cDNA preparation. The cDNA libraries were constructed and sequenced using an Illumina HiSeq™ 2000 located at the Beijing Genomics Institute (BGI, Shenzhen, China; http://www.genomics.cn/index). The data were analyzed according to the method described by Ren et al. (2014). Briefly, clean reads were obtained by removing adaptor sequences, reads in which the percentage of unknown nucleotides (N) was greater than 5% and low quality reads (The rate of reads which quality value ≤ 10 is more than 20%). The clean reads were then assembled into Unigenes using the Trinity software (Grabherr et al., 2011). Finally, blastx alignment (*e* < 0.00001) between Unigenes and protein databases like NR, Swiss-Prot, KEGG and COG were performed, and the best aligning results are used to decide sequence direction of Unigenes.

### Identification of DEGs related to amino acid metabolism and secondary metabolism

Unigene expression was calculated using the FPKM method, and the differentially expressed genes (DEGs) were identified according to stringent criteria: a *P* < 0.05, an FDR ≤ 0.001 and a |log_2_Ratio| ≥ 1.0. The DEGs were then subjected to KEGG Ontology (KO) enrichment analysis based on a hypergeometric test. In addition, DEGs related to the metabolism of major amino acids (e.g., theanine, glutamate, and alanine) and secondary metabolites (e.g., catechins, flavone, and caffeine metabolism) were identified from KEGG annotation, and a hierarchical clustering analysis was then carried out using Cluster 3.0 software. To investigate the molecular mechanisms of drought stress affecting the accumulation of main bioactive ingredients, DEGs related to three major metabolic pathways in *C. sinensis*, flavonoids, caffeine and theanine biosynthesis pathway, were selected for more detailed analyses.

### Quantitative real-time PCR (qRT-PCR) analysis of the selected DEGs

qRT-PCR was used to confirm the accuracy of the differential expression of DEGs via RNA-Seq. Total RNA was isolated from the leaf samples and treated with DNase I to remove any genomic DNA contamination. The single-stranded cDNAs used for real-time PCR analysis were synthesized using a PrimeScript^*TM*^ RT Reagent Kit with gDNA Eraser (TaKaRa, Dalian, China). qRT-PCR was carried out using SYBR Premix Ex TaqTM II (TaKaRa, Dalian, China) on an Eppendorf Real-Time PCR System (Mastercycler® ep realplex, Germany) according to the manufacturer's protocol, and the amplification was conducted as described by Ren et al. (2014). The *C. sinensis* β-actin gene (*Cs*β*-actin*, GenBank: HQ420251.1) was amplified as an internal reference standard, and the relative expression levels were calculated using the 2^−ΔΔ*CT*^ method (Livak and Schmittgen, 2001). Three biological and three technical replicates were performed for each sample, and the primers used for qRT-PCR are listed in Supplementary S1.

### Statistical analyses

Each experiment was repeated at least three times, and all data are expressed as the means ± standard deviations (SD). Group differences were tested using one-way ANOVA and Duncan's test, and significant differences among various treatment groups are represented by different letters (*P* < 0.05). The data were analyzed using SPSS 20 software.

## Results

### Changes in total polyphenol, flavonoid, and free amino acid content

**Figure 1:**
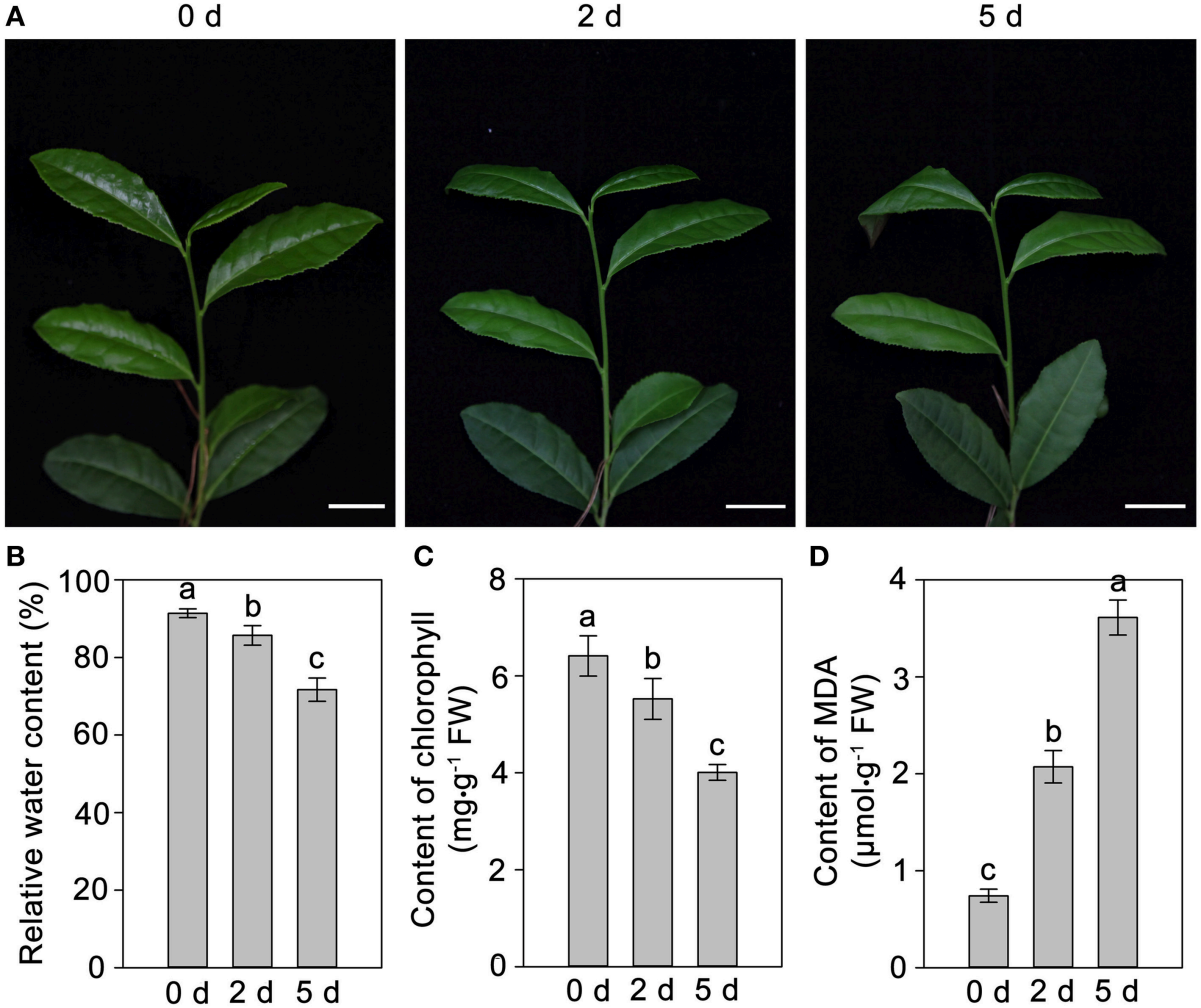
**Effects of drought stress on the phenotype and physiological characteristics of ***C. sinensis*** leaves**. The leaves begun to wilt at 2 days under drought stress, the wilting degree of leaves deepened and exhibited scorch in leaf tips at 5 days **(A)**. The RWC and chlorophyll content in *C. sinensis* leaves decreased rapidly **(B,C)** and the content of MDA increased significantly **(D)** at 2 and 5 days after treated with drought stress.

**Figure 2:**
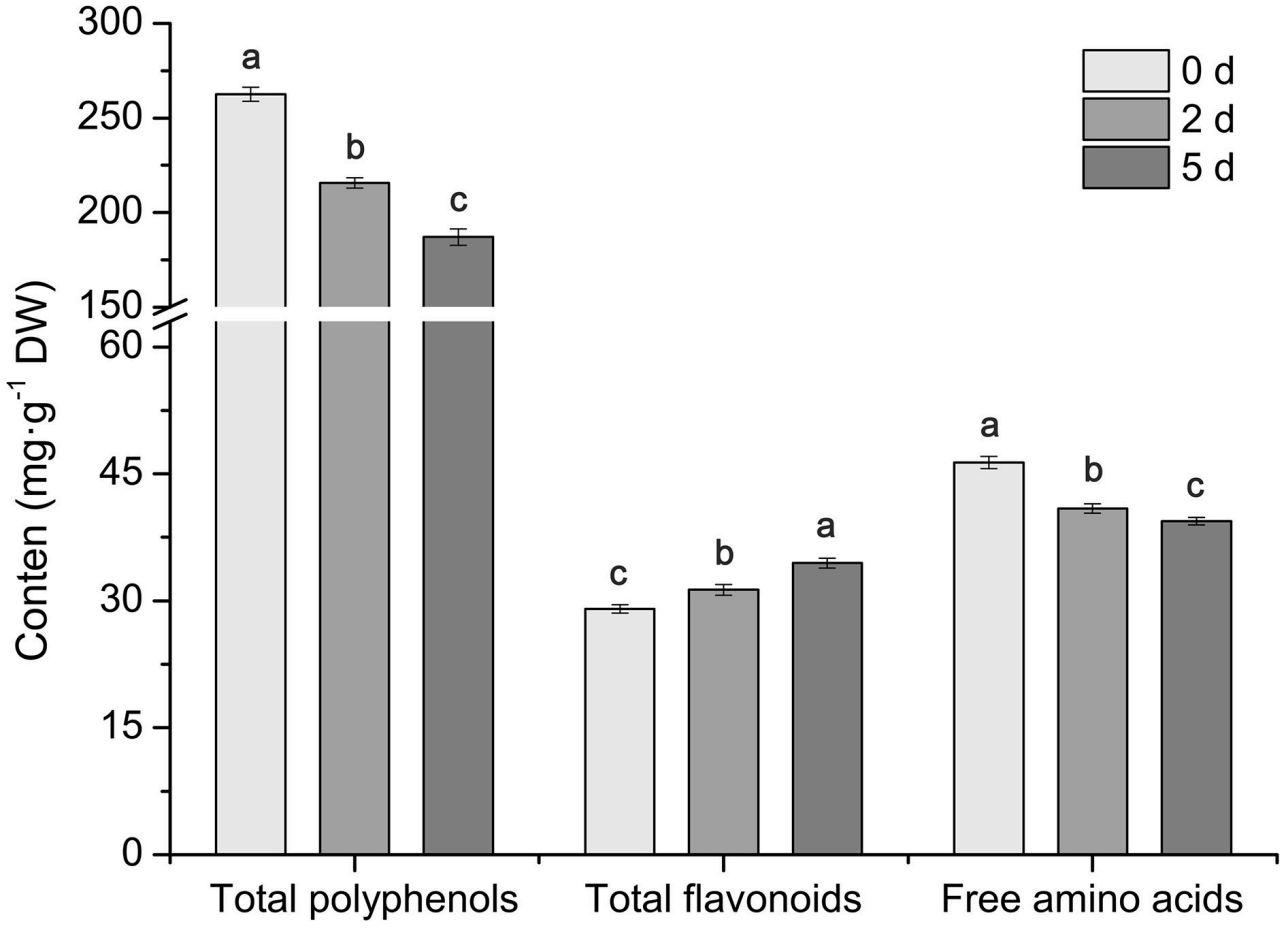
**Effects of drought stress on the total polyphenol, flavonoid and free amino acid content in ***C. sinensis*** leaves**. Drought stress significantly decreased the amount of total polyphenols and free amino acids and increased the total flavonoids. Values are presented as the mean ± SD from three independent experiments, and different letters indicate significant differences at *P* < 0.05 according to Duncan's test.

The phenotypic changes of *C. sinensis* leaves in response to drought stress were recorded at 0, 2, and 5 days. The leaves of *C. sinensis* plants had begun to wilt after 2 days of drought stress, and, the wilting degree of leaves deepened and the leaf tips exhibited scorch at 5 days (Figure 1A). In addition, the analysis of physiological characteristics showed that the RWC and chlorophyll content in *C. sinensis* leaves decreased rapidly (Figures 1B,C) and the content of MDA increased significantly (Figure 1D) at 2 and 5 days after treated with drought stress, implying drought stress obviously affected the normal growth of *C. sinensis* plants, particularly its leaves. Simultaneously, the total polyphenols, flavonoids and free amino acids in the leaf samples were measured after drought stress at different time points (0, 2, and 5 days). As shown in Figure 2, the total polyphenol content significantly and rapidly decreased after drought stress at 2 and 5 days compared with the control plants (0 days). The total free amino acid content showed similar decreases in response to drought stress. Interestingly, drought stress significantly increased the content of total flavonoids.

### The effect of drought stress on catechins and caffeine content

A typical HPLC profile of detected individual catechins and caffeine shows seven individual catechins and caffeine (Supplementary S2), and the changes in the individual catechin components and caffeine in leaf samples after different durations of drought stress are shown in Supplementary S3. The concentrations of individual catechins and caffeine among different time points were compared, as shown in Table 1. Specifically, the amounts of gallocatechin (GC), epigallocatechin (EGC), catechin (Cat), and epicatechin (EC) significantly and gradually decreased after drought stress compared with the control (0 days). Unexpectedly, the concentrations of epigallocatechin gallate (EGCG), gallocatechin gallate (GCG), and epicatechin gallate (ECG) significantly decreased after 2 days of drought stress treatment and then increased slightly at 5 days; however, the overall trends were of decline. In addition, drought stress rapidly reduced the caffeine accumulation in *C. sinensis* leaves.

**Table 1:** **Drought stress-induced changes in the amount of individual catechins and caffeine (mg·g^−1^ DW)**.

**Treatment time (d)**	**GC**	**EGC**	**Cat**	**EC**	**EGCG**	**ECG**	**GCG**	**Caffeine**
0	3.47 ± 0.03a	24.8 ± 0.29a	14.18 ± 1.07a	9.74 ± 0.19a	59.84 ± 1.82a	14.78 ± 0.85a	5.40 ± 0.05a	26.36 ± 0.03a
2	3.20 ± 0.02b	23.24 ± 0.82b	9.52 ± 0.08b	8.43 ± 0.12b	18.94 ± 0.05c	4.07 ± 0.04c	3.81 ± 0.10c	23.15 ± 0.18b
5	3.02 ± 0.02c	19.46 ± 0.10c	10.21 ± 0.11b	8.84 ± 0.08b	36.50 ± 0.44b	10.69 ± 0.11b	4.34 ± 0.06b	17.85 ± 0.25c

*Values are presented as the mean ± SD from three independent experiments, and different letters indicate significant differences at P < 0.05 according to Duncan's test*.

### The effect of drought stress on theanine and other amino acid content

A typical HPLC profile of detected theanine is shown in Supplementary S4, and the changes in the concentration of theanine among different time points were compared, as shown in Supplementary S5. Theanine accumulation significantly and gradually decreased from 30.168 mg·g^−1^ (0 days) to 28.017 mg·g^−1^ (2 days) and 23.989 mg·g^−1^ (5 days; Table 2). Simultaneously, the content of 17 common amino acids was measured. The levels of several amino acids changed to varying degrees after treatment with drought stress (Table 2). Specifically, the content of Glu, Gly, Met, Leu, Phe, Arg, and Lys significantly decreased after drought stress compared with the control (0 days). In contrast, drought stress treatment significantly increased Asp, Ser and Pro accumulation, especially Pro accumulation; this accumulation may be closely related to the drought tolerance of *C. sinensis*. Cys, Val, Ile, Tyr, His, Thr, and Ala did not show marked level changes in response to drought stress.

**Table 2:** **Drought stress-induced changes in content of theanine and other free amino acids (mg·g^−1^ DW)**.

**Amino acid**	**Treatment time (d)**		
	**0**	**2**	**5**
The	30.168 ± 0.471a	28.017 ± 0.411b	23.989 ± 0.411c
Asp	1.238 ± 0.007b	1.224 ± 0.009b	1.259 ± 0.006a
Thr	0.627 ± 0.003a	0.618 ± 0.005b	0.626 ± 0.002a
Ser	0.684 ± 0.003b	0.690 ± 0.009ab	0.698 ± 0.003a
Glu	1.711 ± 0.009a	1.638 ± 0.019b	1.564 ± 0.010c
Gly	0.771 ± 0.007a	0.735 ± 0.006b	0.717 ± 0.005c
Ala	0.776 ± 0.006a	0.757 ± 0.006b	0.783 ± 0.005a
Cys	0.136 ± 0.004a	0.134 ± 0.002a	0.139 ± 0.003a
Val	0.763 ± 0.004ab	0.755 ± 0.004b	0.765 ± 0.006a
Met	0.162 ± 0.001a	0.153 ± 0.004b	0.153 ± 0.003b
Ile	0.597 ± 0.003ab	0.591 ± 0.003b	0.602 ± 0.004a
Leu	1.203 ± 0.004a	1.176 ± 0.006c	1.188 ± 0.006b
Tyr	0.397 ± 0.007a	0.391 ± 0.007a	0.395 ± 0.006a
Phe	0.777 ± 0.001a	0.758 ± 0.008b	0.743 ± 0.005c
Lys	0.996 ± 0.007a	0.991 ± 0.006ab	0.981 ± 0.005b
His	0.322 ± 0.001a	0.322 ± 0.002a	0.323 ± 0.003a
Arg	0.705 ± 0.006a	0.691 ± 0.006b	0.693 ± 0.005b
Pro	0.649 ± 0.003c	0.740 ± 0.005b	0.853 ± 0.009b

*Values are presented as the mean ± SD from three independent experiments, and different letters indicate significant differences at P < 0.05 according to Duncan's test*.

### DEGs related to amino acid metabolism and secondary metabolism

**Figure 3:**
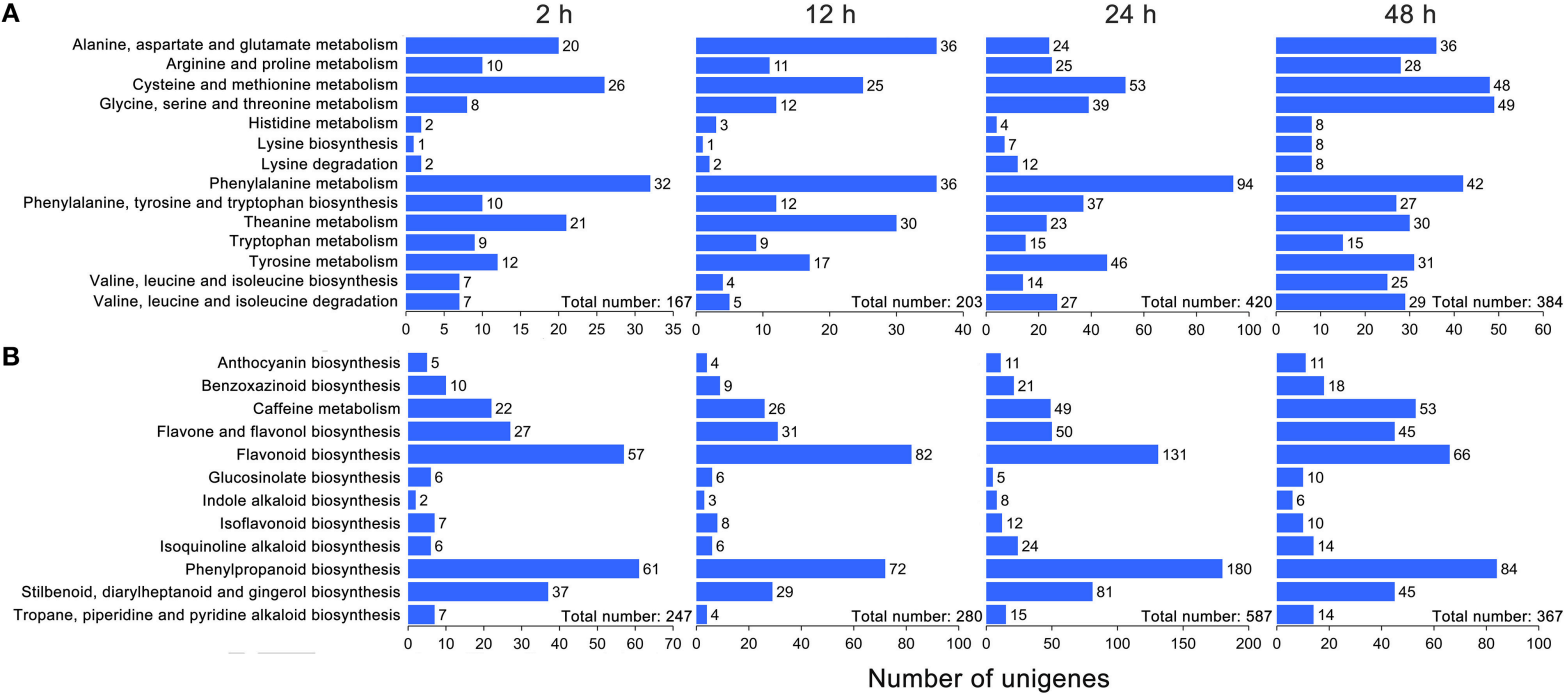
**KEGG ontology (KO) enrichment analysis of DEGs related to amino acid metabolism and secondary metabolism in ***C. sinensis*** leaves under drought stress**. Five hundred and seventy-two and 661 DEGs were annotated and found to be associated with amino acid metabolism **(A)** and secondary metabolism **(B)**, respectively, based on the KEGG database.

**Figure 4:**
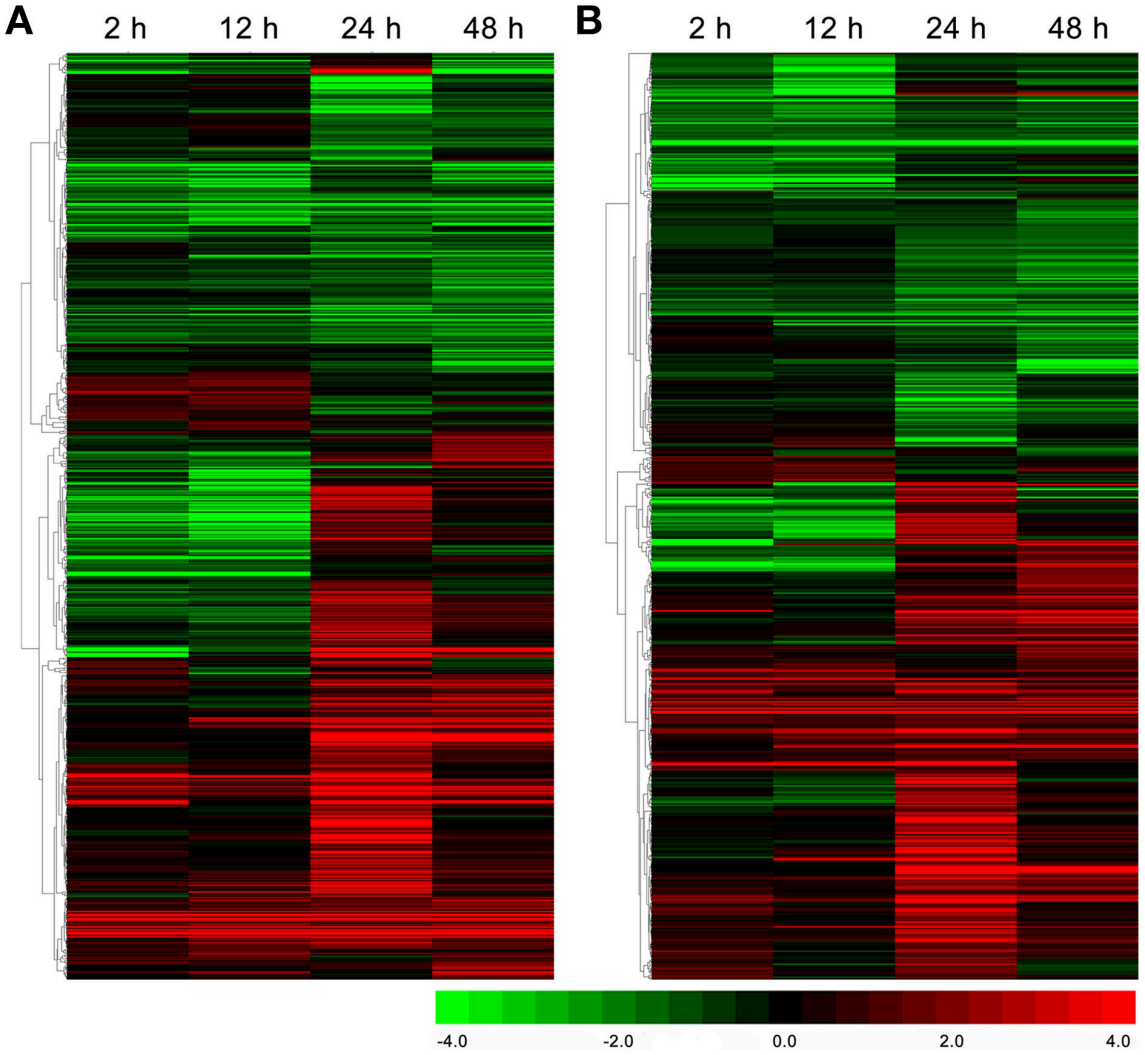
**Hierarchical clustering analysis of the relative expression levels of the DEGs related to amino acid metabolism (A) and secondary metabolism (B) in ***C. sinensis*** leaves under drought stress**. The FPKM ratio of unigene expression is represented on a logarithmic scale for each treatment period (2, 12, 24, or 48 h) and the control (0 h). Red indicates that a gene was up-regulated at that stage, whereas green indicates down-regulated expression.

Based on the KEGG database, 572 and 661 DEGs were annotated and found to be associated with amino acid metabolism and secondary metabolism pathways, respectively (Supplementary S6, S7). As shown in Figure 3A, the total number of DEGs related to various amino acid metabolic pathways increased strongly and reached 420 at 24 h under drought stress; thereafter, it decreased to 384 at 48 h. Interestingly, the number of DEGs related to the phenylalanine metabolic pathway, a key regulatory pathway in the metabolism of the main chemical components in *C. sinensis* leaves, exhibited most obviously (Figure 3A). Similar DEGs change pattern were also observed for the total number of DEGs associated with the metabolic pathways of various secondary metabolites, particularly the key regulatory genes of flavonoid and phenylpropanoid biosynthesis pathways, which affect tea quality by regulating the metabolism of polyphenols and aromatic substances (Figure 3B). In addition, the hierarchical clustering analysis showed that strong changes in DEG expression levels were observed at 24 h of drought stress (Figure 4), implying that 24 h may be an important time point in the regulation of genes related to amino acid metabolism and secondary metabolism; this interpretation is consistent with the changes observed over time in the total number of DEGs.

### Changes in the flavonoid biosynthesis pathway in *C. sinensis*in response to drought stress

**Figure 5:**
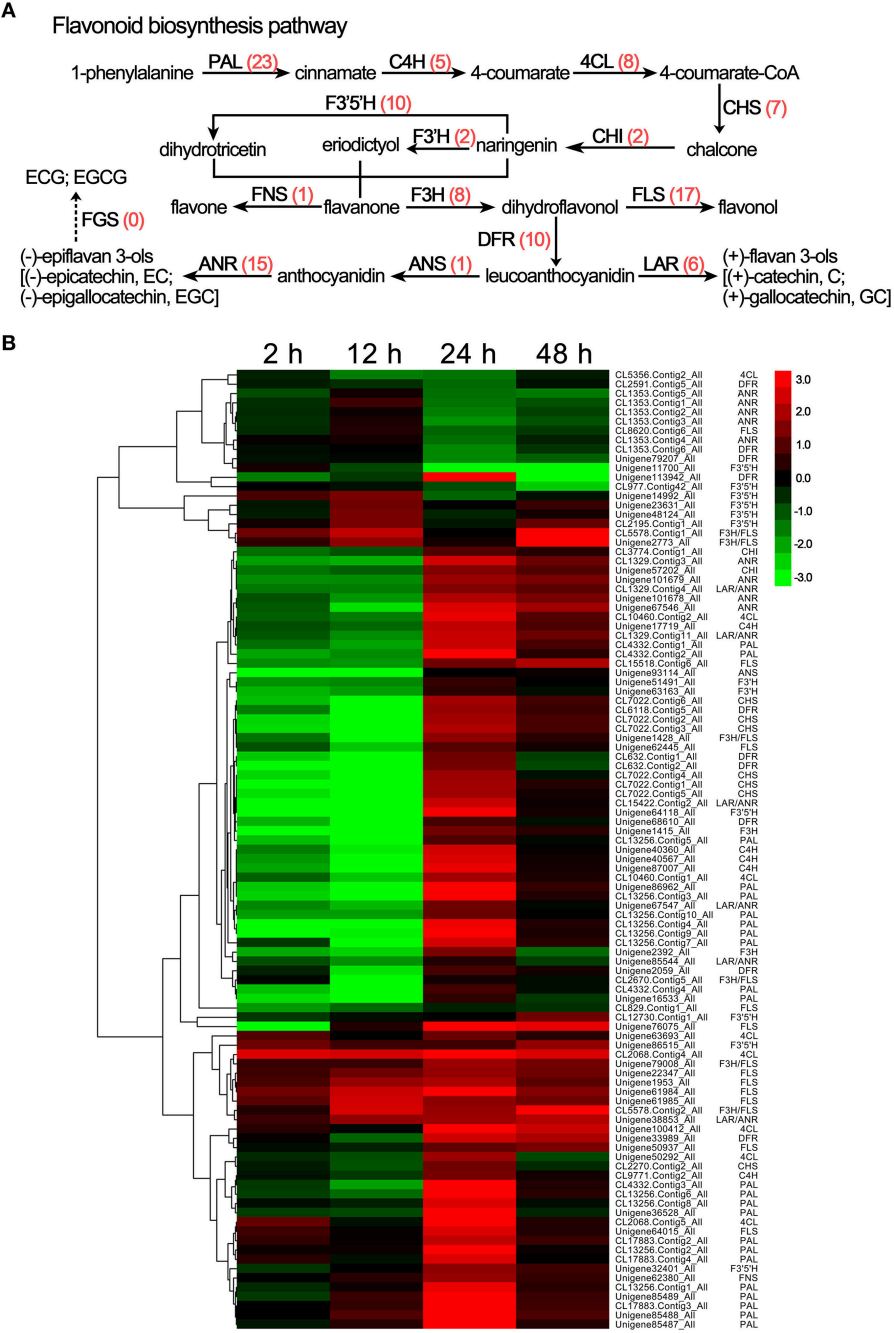
**DEGs involved in flavonoid biosynthesis in ***C. sinensis*** leaves under drought stress. (A)** The flavonoid biosynthesis pathway. The red numbers in parentheses following each gene name indicate the number of corresponding DEGs. PAL, phenylalanine ammonia lyase; C4H, cinnamate 4-hydroxylase; 4CL, 4-coumarate CoA ligase; CHS, chalcone synthase; CHI, chalcone isomerase; F3H, flavanone 3-hydroxylase; F3′5′H, flavonoid 3′,5′-hydroxylase; F3′H, flavonoid 3′-hydroxylase; FLS, flavonol synthase; FNS, flavone synthase; DFR, dihydroflavonol 4-reductase; ANS, anthocyanidin synthase; ANR, anthocyanidin reductase; LAR, leucoanthocyanidin reductase; FGS, flavan-3-ol gallate synthase. **(B)** Hierarchical clustering analysis of the relative expression levels of the DEGs related to flavonoid biosynthesis. The FPKM ratio of unigene expression is represented on a logarithmic scale for each treatment period (2, 12, 24, or 48 h) and the control (0 h). Red indicates that a gene was up-regulated at that stage, whereas green indicates down-regulated expression.

Polyphenols are the most important class of secondary metabolites in *C. sinensis* that includes flavan-3-ols (catechins), flavones, flavonols, isoflavones, flavanones, dihydroflavonols, and anthocyanidins synthesized by the flavonoid biosynthesis pathway, which is one of the most characterized secondary metabolic routes in plant systems (Yang et al., 2015). Here, the transcriptome analysis identified 103 DEGs involved in the flavonoid biosynthesis pathway, and the details of these DEGs are listed in Supplementary S8. As shown in Figure 5A, almost all of the known genes related to the flavonoid biosynthesis pathway were detected under drought stress, such as phenylalanine ammonia lyase (PAL, 23 unigenes), cinnamate 4-hydroxylase (C4H, 5 unigenes), 4-coumarate CoA ligase (4CL, 8 unigenes), chalcone synthase (CHS, 7 unigenes), chalcone isomerase (CHI, 2 unigenes), flavonoid 3′-hydroxylase (F3′H, 2 unigenes), flavonoid 3′, 5′-hydroxylase (F3′5′H, 10 unigenes), flavanone 3-hydroxylase (F3H, 8 unigenes), flavonol synthase (FLS, 17 unigenes), flavone synthase (FNS, 1 unigene), dihydroflavonol 4-reductase (DFR, 10 unigenes), leucoanthocyanidin reductase (LAR, 6 unigenes), anthocyanidin synthase (ANS, 1 unigene), and anthocyanidin reductase (ANR, 15 unigene). In addition, the hierarchical clustering analysis showed that the FLS and FNS unigenes were continuously up-regulated under drought stress (Figure 5B), which may have been responsible for the increases in the total flavonoid content in response to drought stress (Figure 2). Interestingly, the expression levels of most unigenes, such as CHS, DFR, LAR, ANS, and ANR, tended to first decrease and then increase in response to drought stress; this pattern is consistent with the results of the HPLC analyses of the ECG, EGCG, and GCG content (Figure 5B and Table 1).

### Changes in the caffeine biosynthesis pathway in *C. sinensis* under drought stress

**Figure 6:**
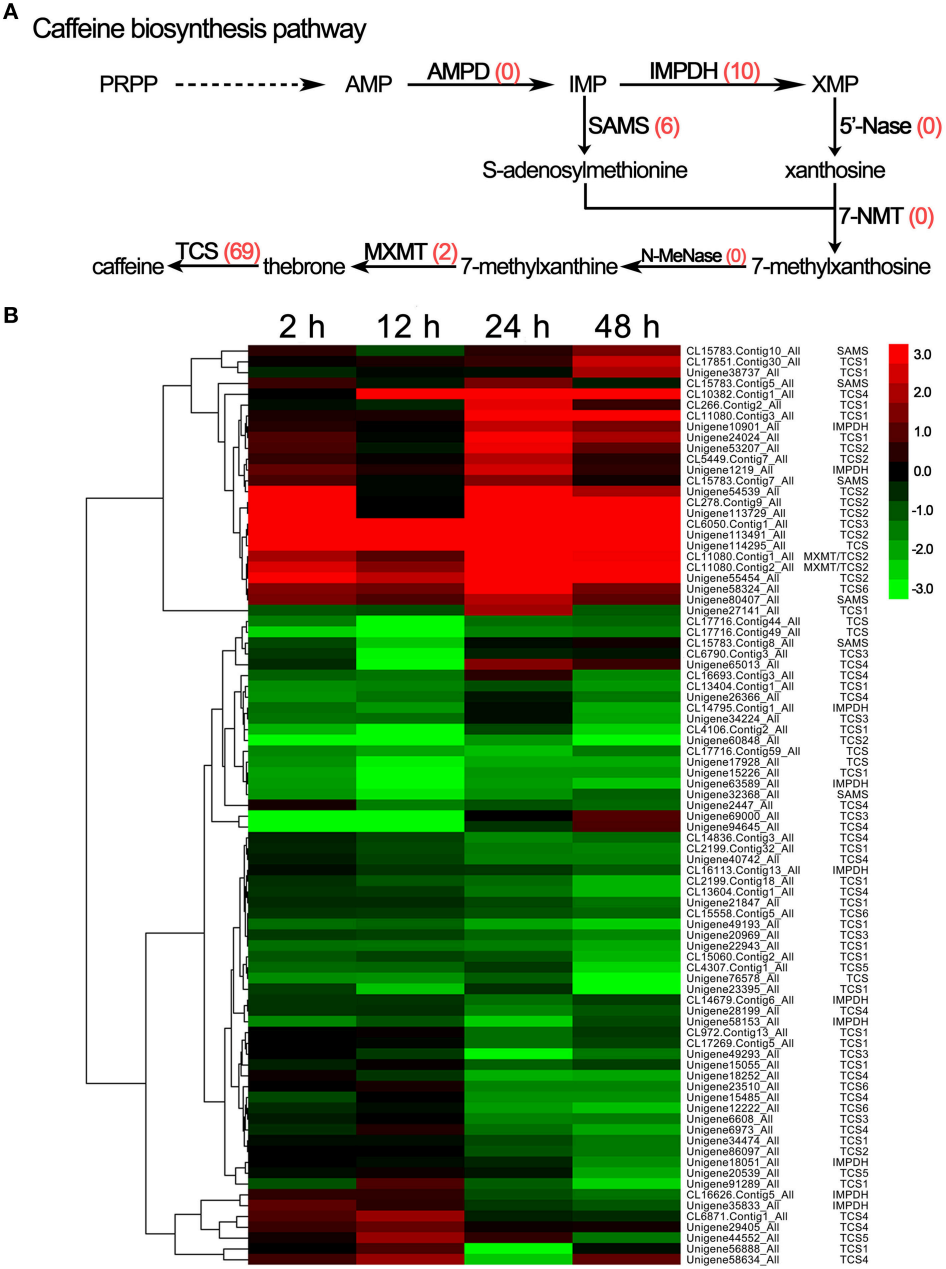
**DEGs involved in caffeine biosynthesis in ***C. sinensis*** leaves under drought stress. (A)** The caffeine biosynthesis pathway. The red numbers in parentheses following each gene name indicate the number of corresponding DEGs. AMPD, AMP deaminase; IMPDH, IMP dehydrogenase; 5′-Nase, 5′-nucleotidase; SAMS, S-adenosylmethionine synthase; 7-NMT, 7-methylxanthosine synthase; N-MeNase, N-methylnucleotidase; MXMT, theobromine synthase; TCS, tea caffeine synthase. **(B)** Hierarchical clustering analysis of the relative expression levels of the DEGs related to caffeine biosynthetic. The FPKM ratio of unigene expression is represented on a logarithmic scale for each treatment period (2, 12, 24, or 48 h) and the control (0 h). Red indicates that a gene was up-regulated at that stage, whereas green indicates down-regulated expression.

The transcriptome analysis identified 85 DEGs related to caffeine, including IMP dehydrogenase (IMPDH, 10 unigenes), S-adenosylmethionine synthase (SAMS, 6 unigenes), theobromine synthase (MXMT, 2 unigenes), and tea caffeine synthase (TCS, 69 unigenes; Figure 6A); the unigene IDs of these genes are listed in Supplementary S9. Simultaneously, the hierarchical clustering analysis showed that most members of the TCS gene family, such as the TCS1, TCS3, TCS4, TCS5, and TCS6 unigenes, were down-regulated in response to drought stress, but a few family members, predominantly TCS2 unigenes, were up-regulated (Figure 6B). In addition, AMP deaminase (AMPD), N-methylnucleotidase (N-MeNase), and 7-methylxanthosine synthase (7-NMT) were not among the DEGs associated with drought stress (Figure 6A).

### Changes in the theanine biosynthesis pathway in *C. sinensis* under drought stress

**Figure 7:**
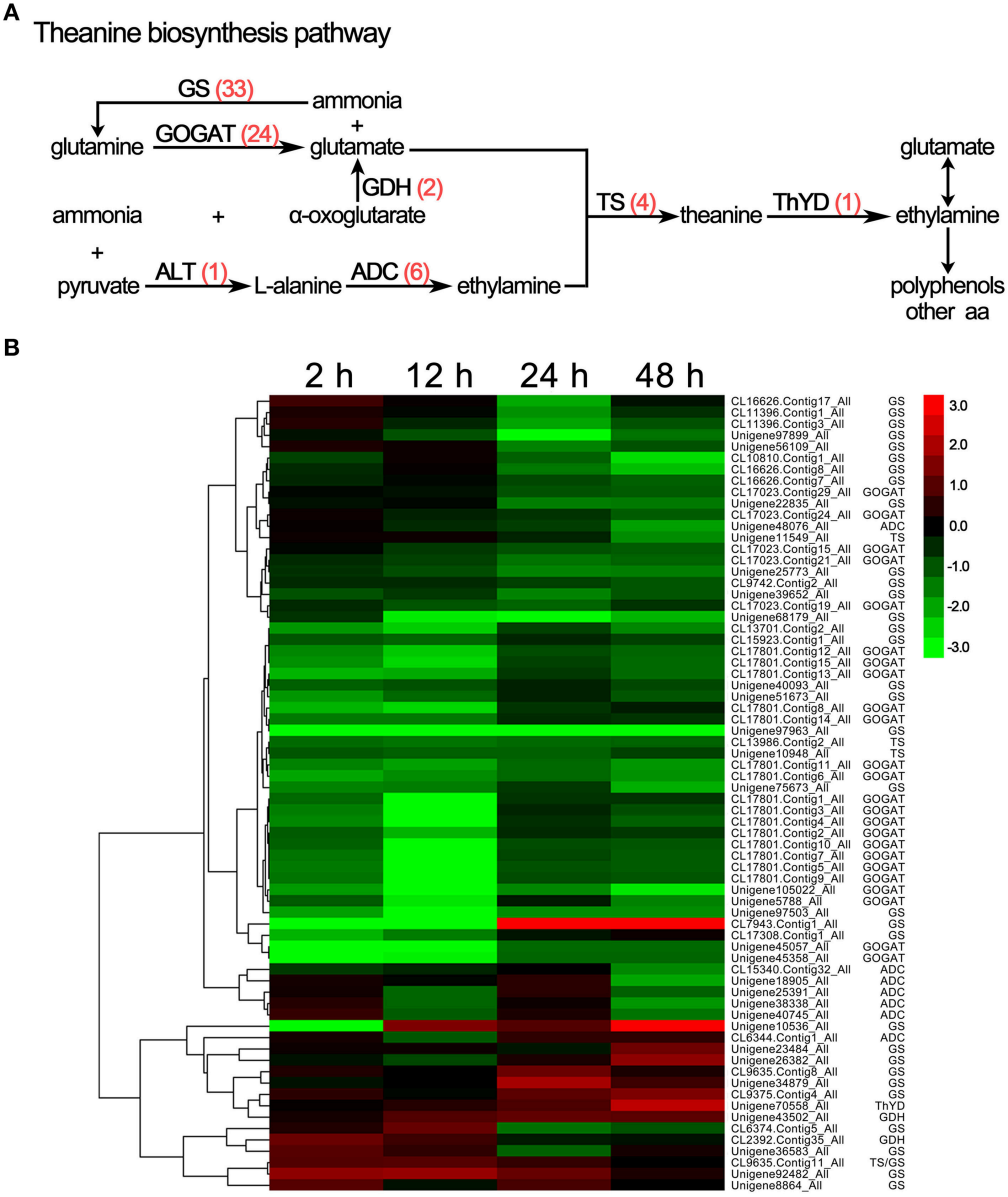
**DEGs involved in theanine biosynthesis in ***C. sinensis*** leaves under drought stress. (A)** The theanine biosynthesis pathway. The red numbers in parentheses following each gene name indicate the number of corresponding DEGs. GS, glutamine synthetase; GOGAT, glutamate synthase; GDH, glutamate dehydrogenase; ALT, alanine aminotransferase; ADC, arginine decarboxylase; TS, theanine synthetase; ThYD, theanine hydrolase. **(B)** Hierarchical clustering analysis of the relative expression levels of the DEGs related to theanine biosynthetic. The FPKM ratio of unigene expression is represented on a logarithmic scale for each treatment period (2, 12, 24, or 48 h) and the control (0 h). Red indicates that a gene was up-regulated at that stage, whereas green indicates down-regulated expression.

Theanine is a unique, non-protein-derived amino acid in the *C. sinensis* plant and is important in the production of the distinctive aroma and umami flavor of tea. As shown in Figure 7A and Supplementary S10, 70 genes related to theanine biosynthesis and degradation were differentially expressed under drought stress, including glutamine synthetase (GS, 33 unigenes), glutamate synthase (GOGAT, 24 unigenes), glutamate dehydrogenase (GDH, 2 unigenes), alanine aminotransferase (ALT, 1 unigene), arginine decarboxylase (ADC, 6 unigenes), theanine synthetase (TS, 4 unigenes), and theanine hydrolase (ThYD, 1 unigenes). Except for ThYD and one GS unigene, all of the unigenes were down-regulated in response to drought stress (Figure 7B), which is consistent with the observed decrease in theanine accumulation in response to drought stress.

### Quantitative real-time PCR (qRT-PCR) validation of DEGs from RNA-Seq

**Figure 8:**
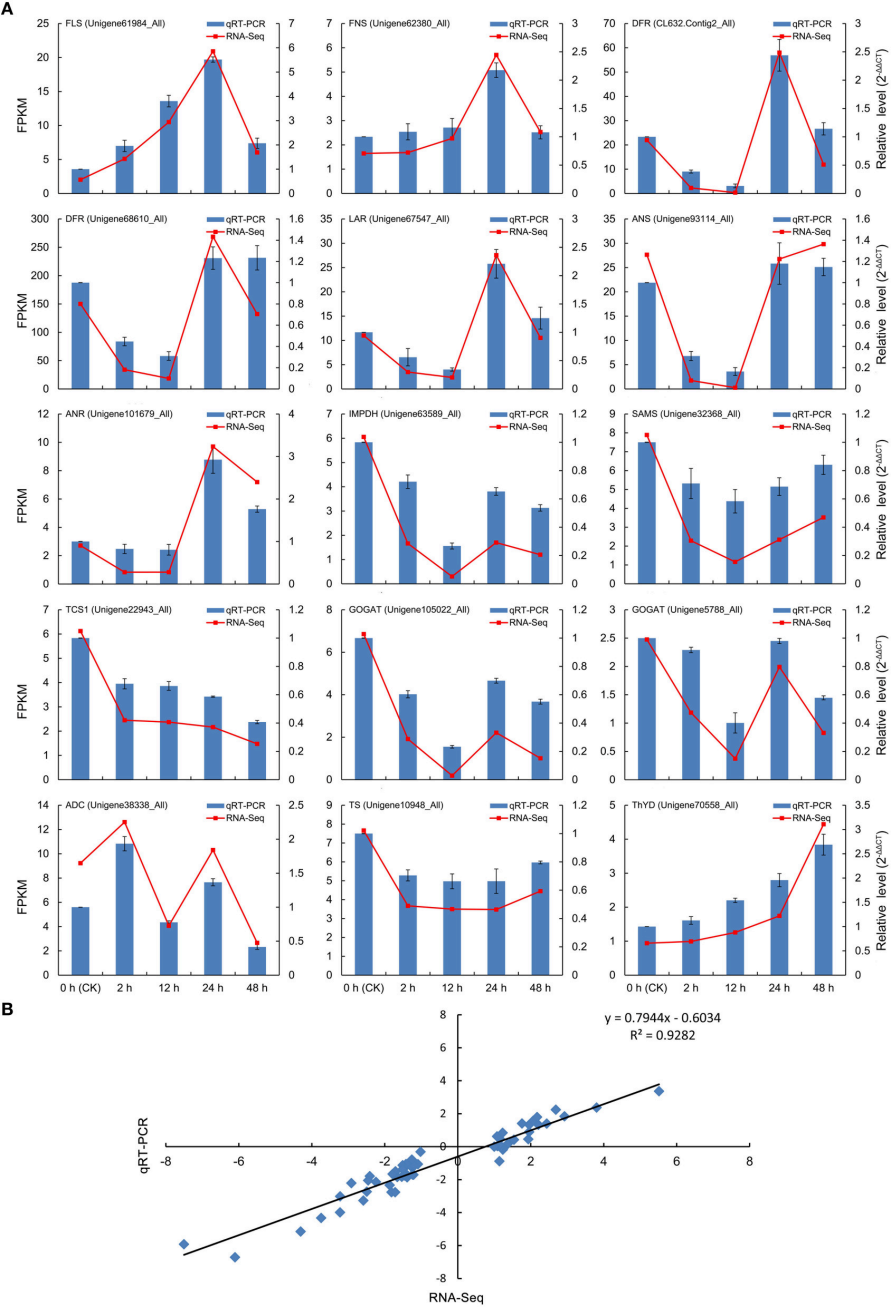
**Verification of the relative expression levels of DEGs by qRT-PCR. (A)** Expression patterns of 15 DEGs related to the flavonoid, caffeine, and theanine biosynthetic pathways by qRT-PCR (blue bar) and RNA-Seq (red line). **(B)** Correlation of the expression levels of the 15 DEGs measured by qRT-PCR and RNA-Seq.

To experimentally validate the expression profiles of unigenes obtained from the Illumina RNA-Seq analysis, 15 DEGs related to flavonoid (7 DEGs), caffeine (3 DEGs), and theanine (5 DEGs) biosynthesis were selected for qRT-PCR, including FLS (Unigene61984_All), FNS (Unigene62380_All), DFR (CL632.Contig2_All and Unigene68610_All), LAR (Unigene67547_All), ANS (Unigene93114_All), ANR (Unigene101679_All), IMPDH (Unigene63589_All), SAMS (Unigene32368_All), TCS (Unigene22943_All), GOGAT (Unigene105022_All and Unigene5788_All), ADC (Unigene38338_All), TS (Unigene10948_All), and ThYD (Unigene70558_All). The qRT-PCR outcomes in each case closely corresponded to the transcript levels estimated from the RNA-Seq output (Figure 8A). In addition, correlation analysis also showed that the expression tendency of these genes from qRT-PCR showed significant similarity (*R*^2^ = 0.93) with the Illumina RNA-Seq data (Figure 8B), suggesting the reproducibility and accuracy of the RNA-Seq results.

## Discussion

Drought stress significantly limits the yield and quality of tea products in tea-growing countries (Cheruiyot et al., 2010). Therefore, understanding the mechanisms underlying the effects of drought stress on *C. sinensis* is important. Although an increasing number of studies have explored the morphological, physiological and molecular changes that occur in *C. sinensis* plants in response to drought stress (Upadhyaya et al., 2008; Das et al., 2012; Zhou et al., 2014), but few have investigated the effects of drought stress on the accumulation of the main bioactive components in *C. sinensis* leaves (Jeyaramraja et al., 2003; Chen et al., 2010). Our investigation shows that dehydration and wilting of leaves are resulted from drought stress and the normal growth of *C. sinensis* plants was seriously affected by drought stress. Meanwhile, we found that drought stress decreased the total polyphenol and free amino acid content in *C. sinensis* leaves, which is consistent with the findings of Cheruiyot et al. (2007) and Chen et al. (2010), who reported that drought stress reduced *C. sinensis* leaf quality as indicated by a significant decrease in total polyphenol and free amino acid levels. In addition, the total flavonoids content significantly increased under drought stress in the present study, which may be an important reason that drought stress affected the liquor color of tea (Liang et al., 1990). Furthermore, HPLC analysis showed that drought stress significantly reduced the content of catechins, caffeine, theanine and some free amino acids in *C. sinensis* leaves. Together, these results indicate that drought stress reduces the quality of *C. sinensis* leaves by affecting the normal growth of *C. sinensis* plants and changing the accumulation of major bioactive ingredients, such as polyphenols (especially catechins), flavonoids, caffeine, theanine and other free amino acids.

Catechins account for approximately 70% of all polyphenols, which are the primary astringent substances of tea (Liu et al., 2015), in *C. sinensis*. In addition, numerous studies have indicated that catechins exert multiple effects on human health and play important antibacterial, antiviral, anti-radiation, and anti-aging roles (Higdon and Frei, 2003; Ho et al., 2008). Previous studies showed that catechins are abundant in the young leaves and buds of *C. sinensis* plants and include esterified catechins [such as epicatechin gallate (ECG), epigallocatechin gallate (EGCG) and gallocatechin gallate (GCG)] and non-esterified catechins [such as catechin (Cat), epicatechin (EC), gallocatechin (GC), and epigallocatechin (EGC); (Graham, 1992)]. However, the accumulation of catechins in *C. sinensis* is very susceptible to various environmental stresses, such as high ultraviolet radiation (Agati and Tattini, 2010), low temperature (Lillo et al., 2008), and drought stress (Jeyaramraja et al., 2003; Wang et al., 2012). We also found that the individual catechin content significantly decreased in response to drought stress, which is similar to the observations of Bhattacharya et al. (2015). Unexpectedly, the EGCG, GCG and ECG concentrations in *C. sinensis* leaves tended to first decrease and then increase in response to drought stress.

Recently, key genes involved in the regulation of the flavonoid biosynthesis pathway in *C. sinensis* have been further recognized and characterized using RNA-Seq technology, particularly the key regulatory genes of monomeric catechins biosynthesis (Shi et al., 2011; Li et al., 2015a). However, few reports are available on the changes of genes related to catechin biosynthesis in response to environmental stresses in *C. sinensis* (Xiong et al., 2013). Our transcriptome analysis data indicate that almost all known genes related to flavonoid biosynthesis are differentially expressed under drought stress. Particularly, the levels of CHS, DFR, LAR, ANS, and ANR tended to decrease and subsequently increase in response to drought stress, which is consistent with the changes of ECG, EGCG, and GCG levels. These findings suggest that the effects of drought stress on the catechins contents of *C. sinensis* leaves depend on the regulation of genes that participate in flavonoid biosynthesis pathway. In addition, the FLS and FNS unigenes, which participate in flavonoid biosynthesis, were up-regulated in response to drought stress. This finding explains the increases in the total flavonoid content of *C. sinensis* leaves in response to drought stress.

Caffeine (1, 3, 7-trimethylxanthine), another important bioactive ingredient in *C. sinensis* plants, is a purine alkaloid that has been widely used as stimulant or ingredient in drugs (Li et al., 2015a). Caffeine is mainly synthesized in young leaves in *C. sinensis* via a typical caffeine biosynthetic pathway including purine biosynthesis and purine modification steps (Ashihara and Kubota, 1986). In the present study, IMPDH, SAMS, MXMT, and TCS genes were identified, and most of these genes were down-regulated in response to drought stress. This finding is consistent with the decreases in the caffeine content in *C. sinensis* leaves following drought stress. These results reveal that drought stress inhibits the expression of genes related to caffeine biosynthesis, such as IMPDH, SAMS, MXMT, and TCS, thereby reducing the accumulation of caffeine in *C. sinensis* leaves.

Theanine is generally considered to play crucial roles in producing the distinctive aroma and umami flavor of tea. Therefore, theanine content is important in determining the quality of a tea product (Mu et al., 2015). Previous studies have shown that theanine biosynthesis begins with glutamine and pyruvate and includes the downstream GS, GOGAT, GDH, ALT, ADC, and TS in the buds, leaves and roots of *C. sinensis* (Shi et al., 2011; Li et al., 2015a). Our data revealed that the accumulation of theanine was significantly decreased by drought stress in *C. sinensis* leaves and this decrease was accompanied by decreases in expression levels of GOGAT, GDH, ADC, and TS and an increase in the expression of ThYD (a key enzyme gene in the theanine biodegradation pathway). According to these results, we speculate that the decrease of theanine in response to drought stress largely depends on changes in the expression levels of genes related to theanine biosynthesis and biodegradation. Furthermore, the content of Glu, Gly, Met, Leu, Phe, Arg, and Lys significantly decreased in response to drought stress, which results in obvious decrease in the quality of *C. sinensis* leaves (Zhu et al., 2016). Meanwhile, our RNA-Seq analysis identified various DEGs related to amino acid metabolism suggesting there is a potential regulatory mechanism for the changes of above amino acids.

In summary, our results suggest that drought stress significantly reduced the quality of *C. sinensis* leaves, as evidenced by abnormality of the phenotype, physiological characteristics and changes in the content of major bioactive ingredients, such as polyphenols, flavonoids, and free amino acids. In addition, the amounts of catechin, caffeine, theanine and some other amino acids in *C. sinensis* leaves significantly decreased under drought stress, which further confirms the effects of drought stress on leaf quality. Furthermore, we used RNA-Seq technology to identify DEGs related to amino acid metabolism and secondary metabolism in *C. sinensis* leaves under drought stress. Particularly, the key regulatory genes of catechins, caffeine and theanine biosynthesis pathway were differentially expressed, which provide insight into the molecular mechanisms that underlie the events described above. Overall, these data provide further insight into the mechanisms underlying the changes in the accumulation of the main bioactive ingredients that occur in response to drought stress and influence leaf quality in *C. sinensis* plants.

## Author contributions

Conceived and designed the work: WW, XL, YW. Performed the experiments: WW, HX, MW, QM, LW. Analyzed the data: WW, HX, MW, LW, NK. Wrote the paper: WW, QM. Revised the paper critically: XL, YW.

### Conflict of interest statement

The authors declare that the research was conducted in the absence of any commercial or financial relationships that could be construed as a potential conflict of interest.
