# Supplementary figures and images for: Transcriptomic Analysis Reveals the Molecular Mechanisms of Drought-Stress-Induced Decreases in Camellia sinensis Leaf Quality
Source: Front Plant Sci. 2016 Mar 30;7:385. doi: 10.3389/fpls.2016.00385 (PMC4811933; doi:10.3389/fpls.2016.00385)

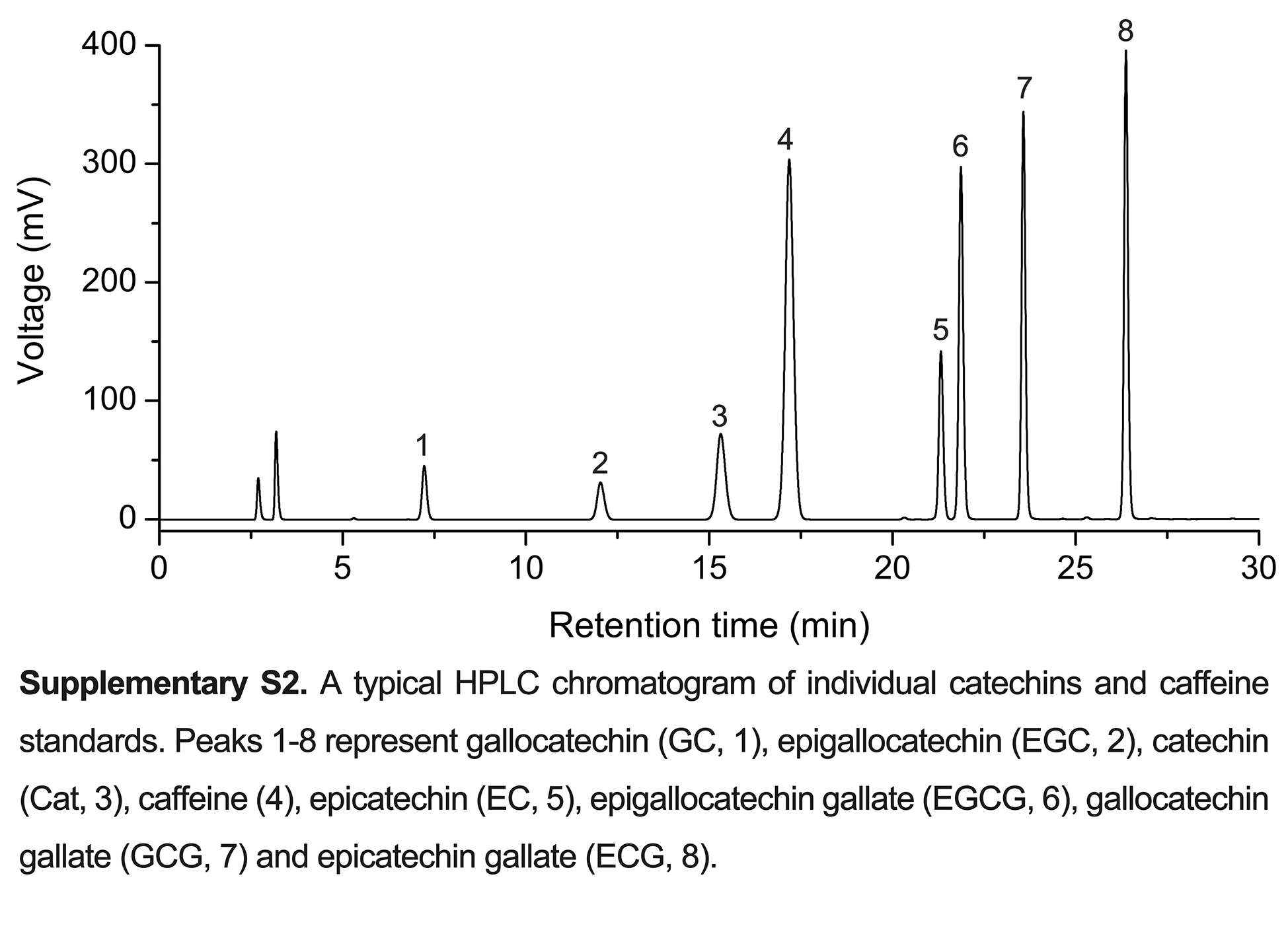

Supplement: Supplementary file 7 [file Image1.TIF]

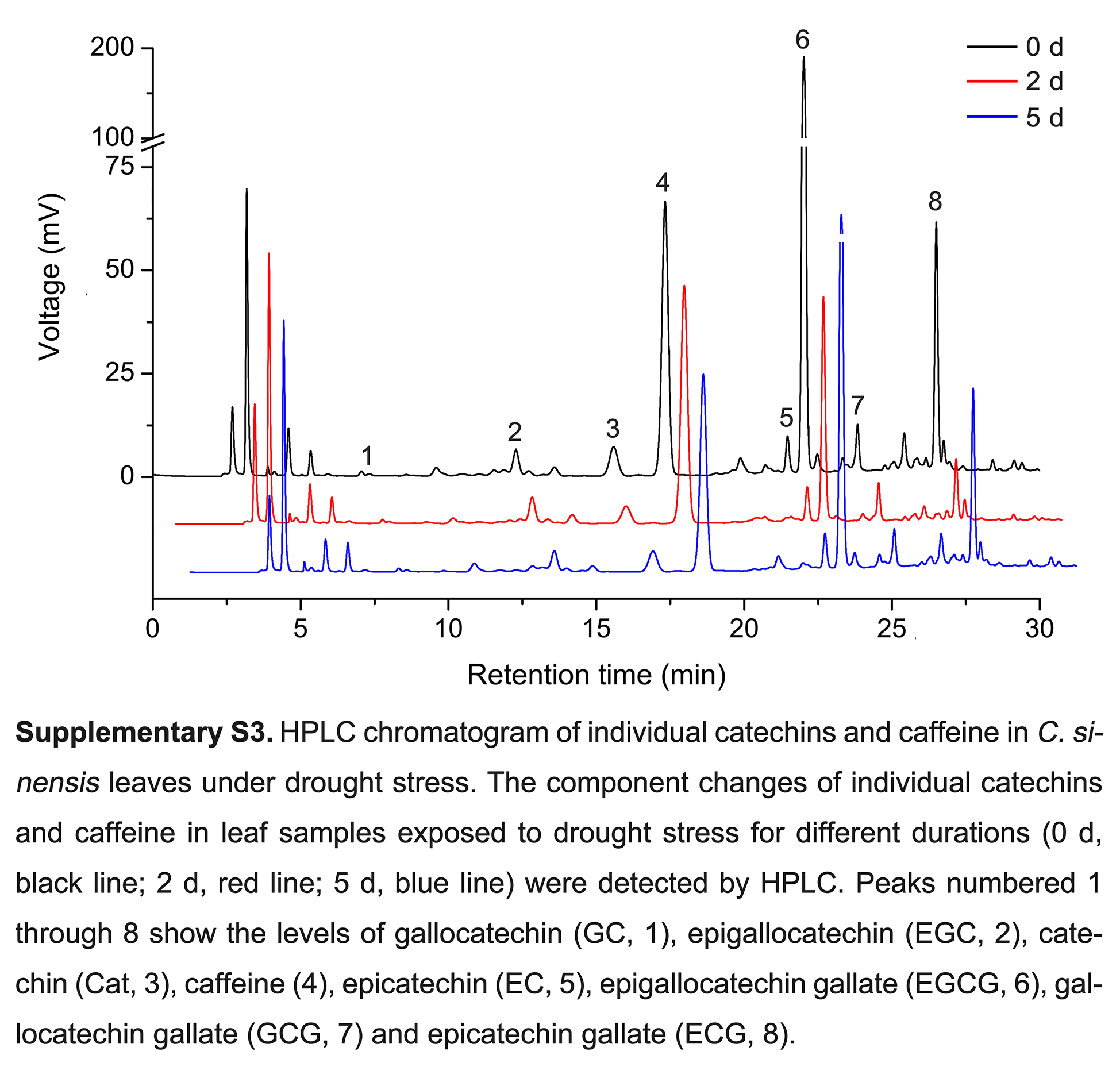

Supplement: Supplementary file 8 [file Image2.TIF]

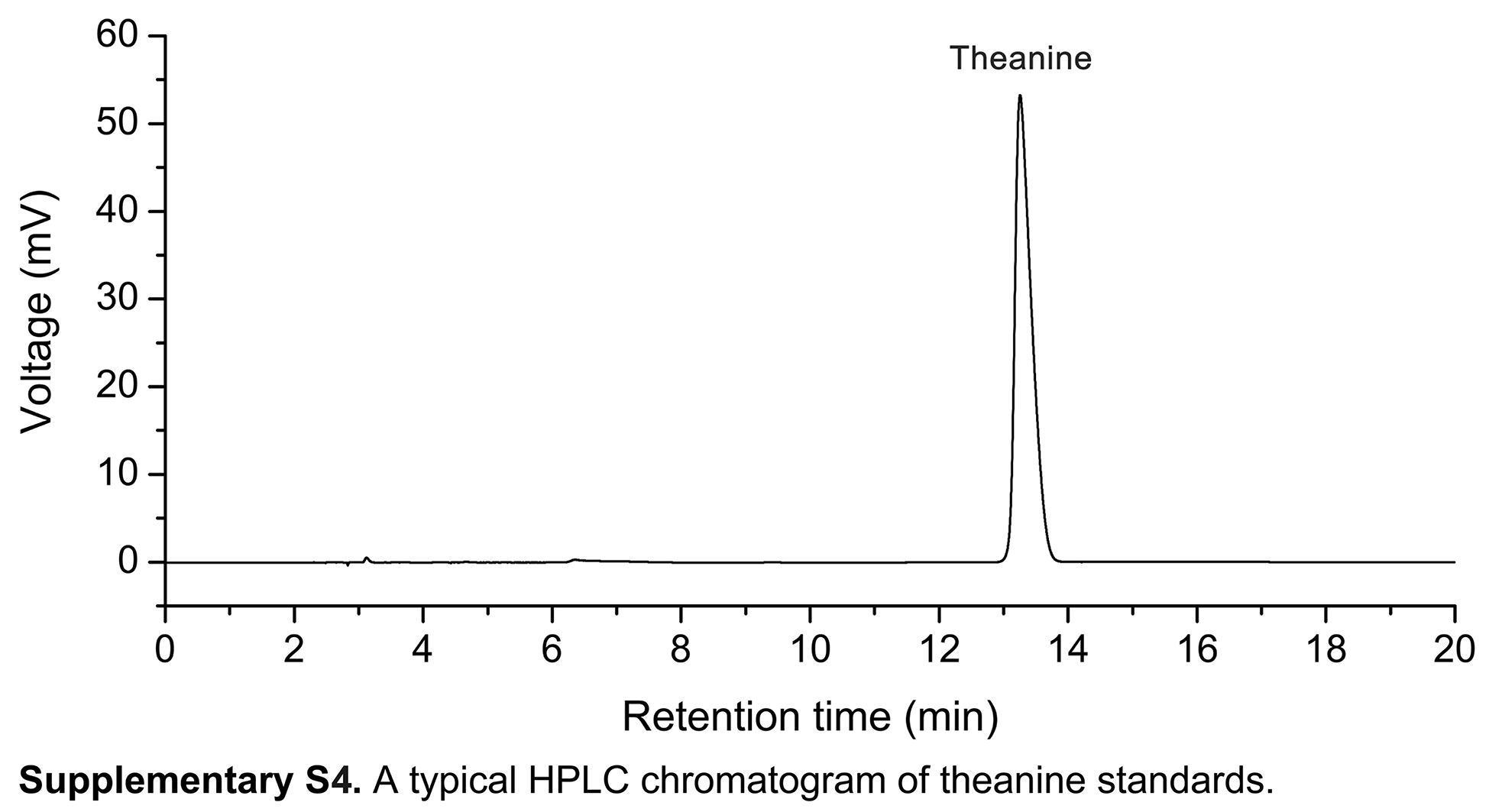

Supplement: Supplementary file 9 [file Image3.TIF]

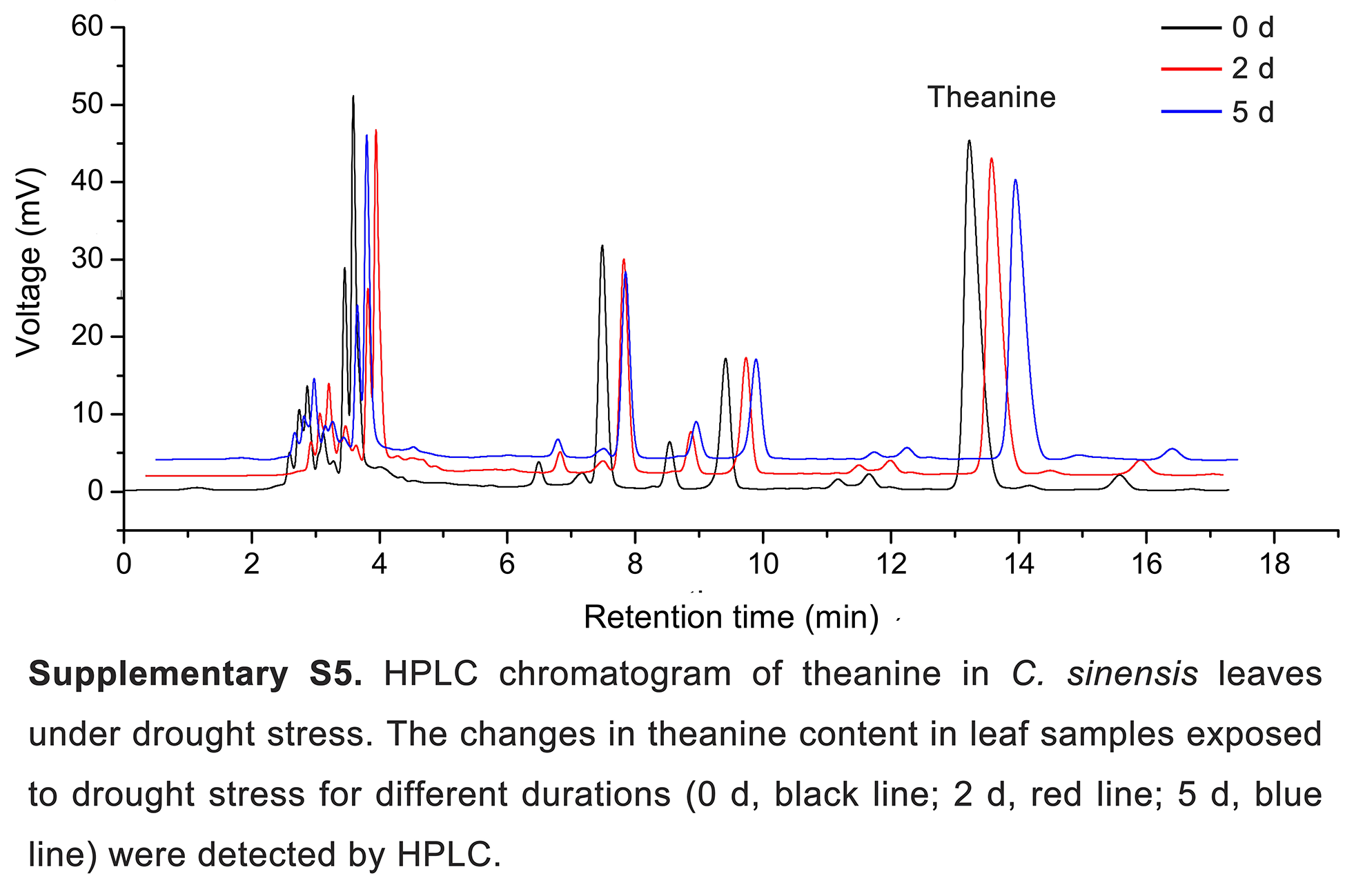

Supplement: Supplementary file 10 [file Image4.TIF]
